# Calibration-Free Single-Anchor Indoor Localization Using an ESPAR Antenna

**DOI:** 10.3390/s21103431

**Published:** 2021-05-14

**Authors:** Mateusz Groth, Krzysztof Nyka, Lukasz Kulas

**Affiliations:** Department of Microwave and Antenna Engineering, Faculty of Electronics, Telecommunications and Informatics, Gdansk University of Technology, Narutowicza 11/12, 80-233 Gdansk, Poland; krznyka@pg.edu.pl (K.N.); lukasz.kulas@pg.edu.pl (L.K.)

**Keywords:** Internet of Things (IoT), wireless sensor network (WSN), switched-beam antenna, electronically steerable parasitic array radiator (ESPAR) antenna, indoor positioning, received signal strength (RSS), fingerprinting

## Abstract

In this paper, we present a novel, low-cost approach to indoor localization that is capable of performing localization processes in real indoor environments and does not require calibration or recalibration procedures. To this end, we propose a single-anchor architecture and design based on an electronically steerable parasitic array radiator (ESPAR) antenna and Nordic Semiconductor nRF52840 utilizing Bluetooth Low Energy (BLE) protocol. The proposed algorithm relies on received signal strength (RSS) values measured by the receiver equipped with the ESPAR antenna for every considered antenna radiation pattern. The calibration-free concept is achieved by using inexpensive BLE nodes installed in known positions on the walls of the test room and acting as reference nodes for the positioning algorithm. Measurements performed in the indoor environment show that the proposed approach can successfully provide positioning results better than those previously reported for single-anchor ESPAR antenna localization systems employing the classical fingerprinting method and relying on time-consuming calibration procedures.

## 1. Introduction

Positioning and navigation systems play an important role in daily lives, since global navigation satellite system (GNSS) applications cover a number of different location-based services, such as wildlife protection, road applications, and security and safety [1]. Even though GNSS provide reliable positioning outdoors [2], the satellite signals cannot reach indoors [3], so global positioning system (GPS) receivers cannot be applied in indoor environments. Considering that people spend more than 80% of their lifetime indoors [4], indoor positioning is at least as important as outdoor positioning. Additionally, different applications may benefit from indoor positioning, such as marketing and sales [5], health [6], or security and emergency solutions [7]. Thus, a number of indoor positioning technologies have been developed and are currently the subjects of further research. Non-radio technologies such as magnetic [8], visual [9], and inertial [10] technologies can be distinguished. On the other hand, radio technologies include ultra-wideband (UWB) signals [11] and radio frequency (RF) standards, of which the most popular are Wi-Fi and Bluetooth [12], RFID [13], and NFC [14].

Considering RF methods, there are two popular approaches for indoor positioning: geometry mapping and fingerprinting [15]. Due to its popularity, most often 2.4 GHz solutions based on common systems such as 802.11, 802.15.4, or Bluetooth Low Energy (BLE) are utilized. The last technology provides the benefits of relatively inexpensive devices to create positioning systems, as well as being low maintenance due to the long battery life of such devices. Additionally, BLE devices often come with integrated inertial measurement units (IMU), so one can relatively easily implement additional data fusion algorithms relying on IMU sensors [16].

The geometry mapping approach utilizes the estimation of geometric parameters, such as the angle of arrival (AoA) of the signal and the distance of its source with respect to certain reference information [17]. The main drawback of this approach is the potential estimation error that may occur due to the multipath effect in the propagation environment.

Fingerprinting is an approach that minimizes multipath effects by using an offline map of signal features collected in certain locations of the area as a reference to find the best match for the signal received from the unknown position [18]. The advantage of the solution is the relatively low computational power needed to perform the estimation. Nevertheless, in large areas, the calibration phase might be time- and effort-consuming, since the density of the radio map impacts the accuracy of the final estimation. This can be minimized through the utilization of the channel state information (CSI) [19], which includes the amplitude and phase of each orthogonal frequency-division multiplexing (OFDM) channel subcarrier [15] to improve the estimation accuracy in locations outside of the reference points. Unfortunately, this approach requires much higher computational power to perform the signal processing, making the system more expensive, and it is not applicable in BLE.

The fingerprinting approach has two crucial downsides that limit its applicability. First is the aforementioned calibration phase, which requires a large number of precisely measured spatial samples of the signal properties to assure the best positioning results [20]. In dynamic environments, the system needs to be recalibrated regularly as the radio map becomes outdated when the propagation environment changes over time. A number of solutions have been researched to simplify the recalibration process [21,22,23]; nevertheless, in most cases, additional infrastructure is needed. The other drawback is the number of reference nodes that need to be installed on site to collect information about the signal. To some extent, the more reference nodes are used, the more accurate the estimation is, as the received signal strength (RSS) vectors are longer [24]. On the other hand, the number of devices that create the positioning system also affects the final cost of the installation. To solve this issue, single-anchor positioning has been proposed as one of the solutions for simplification and cost reduction in indoor positioning system installations [25,26,27]. In such solutions, instead of many reference modules, only a single device equipped with a reconfigurable antenna able to modify its radiation pattern is used.

In this paper, we present a novel method for calibration-free single-anchor indoor positioning. The solution is evaluated utilizing cost-effective BLE modules as auxiliary reference nodes located in fixed positions on room walls. The nodes act as beacons, transmitting reference signals to be considered during position estimation; in this way, the solution is less sensitive to changes in the environment. Additionally, no human-operated calibration or recalibration is needed, since the system is based on current signals received from the reference nodes. Furthermore, simple BLE beacons have relatively long battery life and low retail cost, thus the use of reference modules does not significantly influence the total cost of the system. As a result, such systems could be particularly valuable in scenarios where regular maintenance is difficult or impossible, including busy organizational units such as hospitals or airports. For the single-anchor localization device, the BLE module together with the energy-efficient electronically steerable parasitic array radiator (ESPAR) antenna were chosen. With the aforementioned antenna, the beam can be controlled by shortening or opening passive elements surrounding the active element in the center.

The resulting novel, calibration-less, single-anchor localization system was tested, providing better results than other systems previously reported in the literature [26,27]. Additionally, the calibration-free approach decreases the risk of accuracy loss over time and reduces the system maintenance costs. According to the authors’ knowledge, there is no other calibration-free indoor positioning system using the single-anchor ESPAR antenna concept. Therefore, the main contribution of this paper is the original approach to single-anchor position estimation by utilizing the angular diversity capabilities of an ESPAR antenna and the reference signals of the reference nodes, providing zero-calibration functionality. Additionally, the proposed approach provides at least 13.7% higher accuracy than available single-anchor approaches relying on time-consuming prior calibration [26,27].

The rest of this paper is organized as follows. In Section 2, the related work is described. Section 3 includes the antenna characterization. Section 4 presents the proposed calibration-free algorithm. The test environment and results are presented in Section 5 and Section 6, respectively, while concluding remarks are presented in Section 7.

## 2. Related Work

An analysis of the parameters of RF signals allows one to estimate the position of a transceiver based on spatial information. To this day, a number of position estimation techniques have been developed [28], however from the perspective of applicability in wireless sensor networks (WSNs), those that do not impose high computation costs and are the easiest to introduce in real scenarios are the most interesting. From this point of view, algorithms that rely on RSS values are the most attractive, as they provide relatively high accuracy while keeping the implementation complexity reasonably low. Consequently, such algorithms can easily be implemented in simple WSN nodes with integrated RSS readouts.

One of the most popular RSS-based localization methods is the trilateration method [29], which is a trigonometric approach where the distances from three access points (APs) of known locations are calculated based on the chosen propagation model. Knowing the positions of the APs and the estimated distances of the mobile nodes from each of them, the positions of the node can be estimated by solving the equation:

(1) $$\{\begin{array}{c} R_{1}=\sqrt{{\left(x-X_{1}\right)}^{2}+{\left(y-Y_{1}\right)}^{2}{\left(z-Z_{1}\right)}^{2}}\\ R_{2}=\sqrt{{\left(x-X_{2}\right)}^{2}+{\left(y-Y_{2}\right)}^{2}{\left(z-Z_{2}\right)}^{2}}\\ R_{3}=\sqrt{{\left(x-X_{3}\right)}^{2}+{\left(y-Y_{3}\right)}^{2}{\left(z-Z_{3}\right)}^{2}} \end{array}$$

where $R_{j}$ is the estimated range from $AP_{j}$ and $X_{j},\;Y_{j},\;Z_{j}$ are the coordinates of $AP_{j}$. The main drawback of this approach is the influence of the environment on the RSS values and the need to use complex propagation models to provide a proper mathematical description of the environment. Similarly, in an approach called centroid localization, *N* closest APs are chosen based on RSS values and the node positions are estimated by calculating the centroid formula [30]:

(2) $$\left(x,y\right)=\left(\frac{X_{1}+X_{2}+\cdots +X_{N}}{N},\frac{Y_{1}+Y_{2}+\cdots +Y_{N}}{N}\right)$$

Another approach to indoor positioning based on the strength of the received signal is called fingerprinting [31]. In this method, two phases can be distinguished, namely offline and online phases. During the offline phase, the system learns the RSS values at the number of defined reference points by collecting measurements across the scene from all of the APs. The values are stored in the database together with the coordinates of each point. During the online phase, the actual position estimation is performed and RSS values are collected and compared with the values in the database. The localization of the device is estimated by calculating the Euclidean distance between the each vector of the RSS values from the offline phase and the ones measured during the online phase, which can be described as:

(3) $$D_{j}=\sqrt{\overset{I}{\underset{i=1}{\sum }}{\left(RSS_{online_{i}}-RSS_{offline_{i,j}}\right)}^{2}}$$

where *I* is the total number of APs for which RSS values are stored, $RSS_{online_{i}}$ is the RSS value measured during the online phase by the *i*th AP, $RSS_{offline_{i,j}}$ is the RSS value for the *i*th AP stored in the database, and *j* is the consecutive number of fingerprints. In the simplest version, to determine the position, the distance is calculated for every reference point and the estimated position is the reference position for which the Euclidean distance has the lowest value. A number of improvements to this method have been presented, such as the arithmetic mean distance from *K* nearest neighbors (KNN) [32] and the weighted mean distance (WKNN).

Indoor positioning based on a single anchor allows one to achieve the localization functionalities using smaller infrastructure in terms of the number of devices. Since UWB signals are more robust to multipath effects [33], to achieve higher estimation accuracy, UWB ranging is often utilized in single anchor positioning. In [34], the authors propose a hybrid approach, which combines UWB ranging modules with data processing from inertial sensors that consist of a 3-axis accelerometer for the step counting and 3-axis magnetometer for the azimuth angle estimation. Additionally, a real-time Kalman filter is used to smooth the ranging results. Inertial measurement data can also be a support for direction of arrival (DoA) and ranging based on typical 2.4 GHz protocols. For example, in [35], the authors present a fusion algorithm for BLE transceiver signal angle and distance estimation based on Kalman filtering and simplified pedestrian dead reckoning (PDR). Angle estimation is achieved with signal-phase measurements on a uniform linear antenna array, while the range is estimated with a path loss model based on RSS. A 10-element uniform linear antenna array was also used for DoA fingerprinting in [36]. In this approach, the angle of arrival is estimated using the minimum variance distortionless response (MVDR) estimator. Position estimation was performed based on a DoA fingerprinting approach using spatial spectrum measurements, where the measured spatial spectrum is compared with the set of training data using the Pearson correlation coefficients. In [37], the authors propose a multipath-based, single-anchor positioning system, which non-coherently acquires directional measurements by exploiting specular multipath components.

Switched-beam antennas are often used for single anchor positioning systems, whereby a single base station in a given area can perform the positioning instead of a number reference nodes, which reduces the deployment cost of the system [25,26,27]. The benefit of such an approach is that it can be integrated with the most popular wireless communication standards such as Wi-Fi and BLE to reduce the number of base stations used as APs. The most promising switched-beam antenna concepts, which can be used in practical single-anchor localization systems, are those that are inexpensive for mass production and easy to integrate with low-cost transceivers, with simple microcontrollers performing RSS measurements and localization estimation [26,38].

Due to the RSS instability and changes in the environment, the radio map for the fingerprinting method deteriorates over time and needs to be updated regularly. Therefore, solutions that simplify or automate the recalibration process give the user the possibility to reduce the maintenance efforts and costs of the system. One such solution was presented in [39], where radio maps for the offline phase of fingerprinting were generated automatically. The authors used IoT Wi-Fi sensors as scanning nodes to analyze the environment. The obtained RSS measurements were then utilized to periodically generate new radio maps using a pre-processed path loss template. The updates were generated by estimating the RSS values for each cell of the 1 m × 1 m grid. In [23], invariant RSS statistics were introduced to eliminate the need for offline recalibration. Particle filters can also be adopted to use crowd-sourced fingerprinting maps for the recalibration [21] and to fuse PDR and positioning estimation data in order to determine and re-estimate the divergence of particle trajectories. These re-estimated trajectories can be adopted to update the radio map. In [22], APs were used to detect permanent changes in RSS values and to modify the radio map accordingly. The update was performed using a Voronoi diagram, excluding from the recalibration those APs for which RSS characteristics changed significantly. Then, the RSS delta was added to each fingerprint as a final stage of recalibration.

Often, the advantages of CSI are harnessed to achieve calibration-free localization. This can be done as described in [40], where the construction of a theoretical CSI fingerprinting database without a site survey was presented. In this approach, the constellation diagram is utilized to represent the relation between the phase difference and AoAs. Theoretical AoAs are derived with respect to existing access points (APs) for any given position and are transformed into the phase difference for a fingerprinting database.

Among other positioning methods, solutions based on product-moment [41] and least-squares [42] correlations between the RSS and the estimated signal strength have been presented. Such methods do not require offline calibration and differ only in the optimization criteria to be calculated; nevertheless, they require assumptions based on the channel propagation models. A further step was taken by the authors of [43], whereby the relation between the RSS and the distance from the APs was used to construct the Voronoi diagram of the area relative to the particular AP locations. During the positioning, the ambiguity region was estimated as the Voronoi cell of the AP of the strongest RSS and was split into two half-planes by analyzing the relative relation between the RSS of each pair of APs. The final position was estimated as the center mass of the final ambiguity region.

## 3. ESPAR Antenna for Single-Anchor Localization

**Figure 1:**
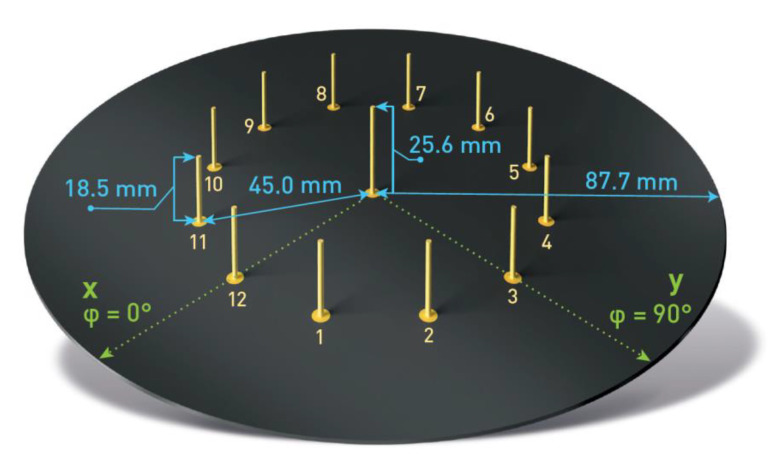
Electronically steerable parasitic array radiator (ESPAR) antenna design.

**Figure 2:**
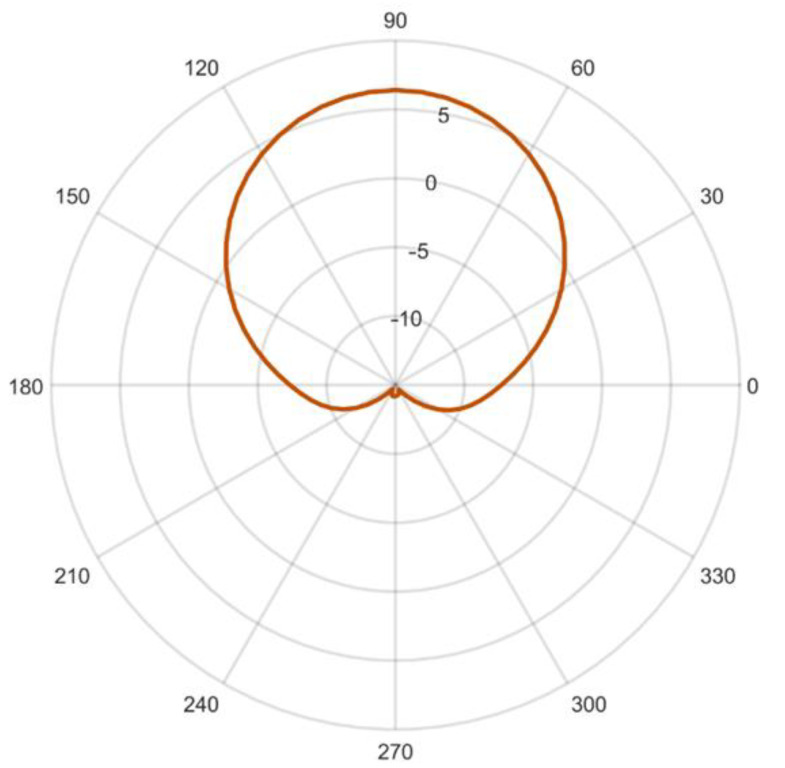
Simulated ESPAR antenna radiation pattern gain (in dBi) at 2.484 GHz for the steering vector $V_{max}^{1}=\left[111110000000\right]$.

The ESPAR antenna considered for the proposed calibration-free indoor positioning system is an interesting, less expensive, more energy-efficient alternative solution to complex antenna arrays [44,45]. Radiation patterns from ESPAR antennae can be modified by changing load impedances connected to passive elements located around the active element. For the proposed calibration-free single-anchor indoor localization method, a simplified concept, as previously presented in [45], was implemented. In this design, as depicted in Figure 1, the antenna consists of 12 passive elements located around the active element, to which the signal output of the transceiver is connected. Each of the 12 passive elements can be shorted or opened through single-pole, double-throw (SPDT) field-effect transistor (FET) switches steered individually from a microcontroller. Shorted passive elements become reflectors while opened ones are directors for the active element. As a result, a directional radiation pattern, as shown in Figure 2, can be created and rotated with a 30° discrete step, forming 12 different directional radiation patterns. The utilization of FET switches brings a significant reduction of power consumption via the antenna circuit as compared to varactor-based solutions [46] and can be successfully used in DoA estimation of battery-powered IoT modules [38,47].

The antenna designed with FEKO electromagnetic simulation software in [45] has a center frequency of 2.484 GHz and directional radiation patterns at 3 dB beamwidth of 73.2°, which can be formed by shorting five consecutive passive elements. Therefore, the antenna configurations can be denoted using the steering vector $V_{\max}^{n}=\left[v_{1}\;v_{2}\cdots v_{s}\cdots v_{11}\;v_{12}\right]$, where $v_{s}=0$ when the *s*th element is shorted and $v_{s}=1$ when it is opened. All of the vectors considered following the steering vector notation and the corresponding radiation patterns are presented in Table 1. It has to be noted that from the localization system perspective, each configuration can be considered as a separate AP for indoor positioning, as each RSS from incoming RF signals for each radiation pattern has a different spatial distribution.

**Figure 3:**
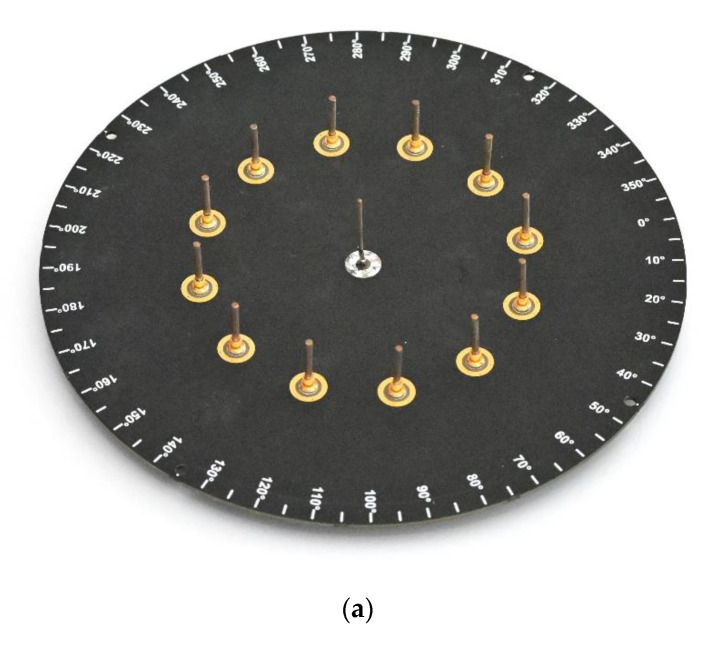

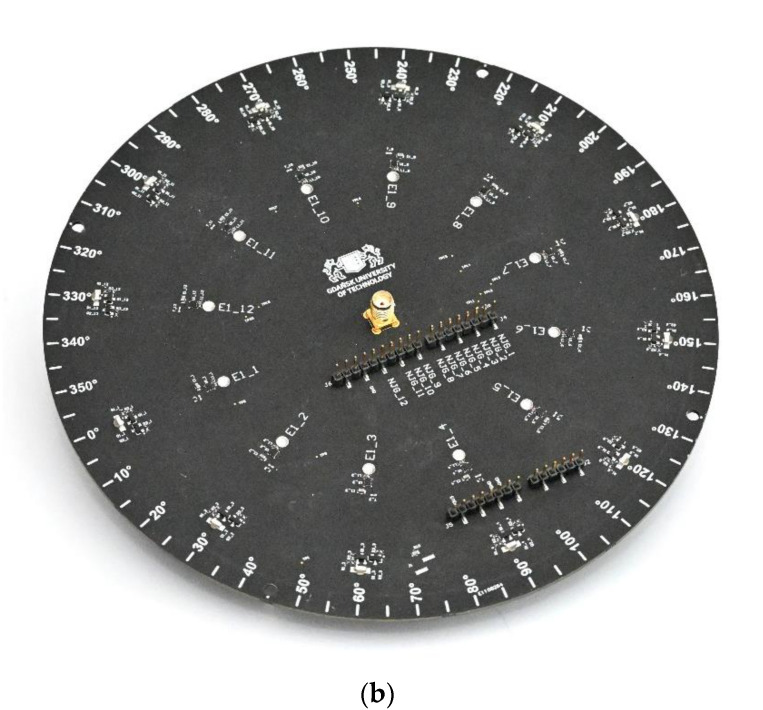
ESPAR antenna: (**a**) top view; (**b**) bottom view.

The antenna was constructed following the described design on 1.55 mm FR4 laminate. The passive elements were constructed with a 2-mm-diameter silver-plated copper rod, while the active element used the same 25.6-mm-long copper rod as an extension of the SMA connector soldered directly to the laminate. To provide beam-switching capability to the antenna, NJG1681MD7 GaAs FET MMICs SPDT switches were chosen. The antenna prototype, as presented in Figure 3, was equipped with Arduino Shield headers for the convenient connection of different WSN modules compatible with the standard. Additionally, 12 LEDs were installed at the bottom of the PCB to represent the status of each passive element. A similar antenna prototype was used in [26] for indoor positioning based on the fingerprinting method.

## 4. Proposed Calibration-Free Algorithm

In this paper, we propose a novel, low-complexity RSS-based method of RF-based indoor positioning using ESPAR antennas that is dedicated to WSN applications. The method integrates aspects of the aforementioned fingerprinting and trilateration approaches and uses active auxiliary reference nodes to decrease the negative influence of indoor RF signal propagation effects, as well as over-time radio map drift. Additionally, by employing the switched-beam ESPAR antenna, the system can implement the single-anchor approach, whereby the RSS measurements and position estimation are performed with only one device, resulting in a significant reduction of the total number of APs in the system. Furthermore, RSS analysis based on beam-switching is much less complex and hardware-independent than direction findings based on phase-shift analysis, which additionally is impossible to implement in certain WSN nodes where IQ samples are not available for the system developer.

The algorithm utilizes simple transmitters as auxiliary reference nodes installed in known positions. The reference nodes transmit signals that are received by the base station (BS) equipped with the ESPAR antenna. For each antenna configuration, the RSS for each reference node is measured and stored in a database. At the same time, the RSS of the localized node is also measured and stored for analysis in the next step. For each reference node, the RSS vector of *j*th device can be denoted as:

(4) $$V_{ref_{j}}=\left[RSS_{ref_{j_{1}}},\;RSS_{ref_{j_{2}}},\;\ldots ,\;RSS_{ref_{j_{12}}}\right]$$

where $RSS_{ref_{j_{i}}}$ is the received signal strength of *j*th node for the *i*th antenna configuration.

The RSS vector for the localized node can be defined by analogy as:

(5) $$V_{loc}=\left[RSS_{loc}{}_{1},\;RSS_{loc}{}_{2},\;\ldots ,\;RSS_{loc}{}_{12}\right]$$

where $RSS_{loc_{i}}$ is the received signal strength for *i*th antenna configuration.

For the localization, two stages can be distinguished. In the first phase, the algorithm looks for the reference nodes closest to the localized node. In order to do this, the Euclidean distance $D_{j}$ between $V_{{ref}_{j}}$ and $V_{loc}$ for each *j*th reference node is calculated as:

(6) $$D_{j}=\|V_{loc}-V_{{ref}_{j}}\|=\sqrt{\overset{12}{\underset{i=1}{\sum }}{\left(RSS_{loc_{i}}-RSS_{ref_{j_{i}}}\right)}^{2}}$$

Since each of the RSS values is measured for different antenna characteristics, the reference nodes for which the calculated distance is the lowest are considered to be in the closest vicinity of the localized node. All of the calculated distances $D_{j}$ can be ordered from the lowest to the highest value:

(7) $$D_{j=k_{1}}\leq D_{j=k_{2}}\leq D_{j=k_{3}}\leq \cdots \leq D_{j=k_{J}}$$

where J is the total number of reference nodes and $k_{1},\ldots ,k_{J}$ are indices of the distances arranged in growing order. Then, for the second phase, K reference nodes for which the calculated distance is the lowest are chosen, so that the actual position can be estimated based on the position of the chosen nodes with indices $\left\{k_{1},\ldots ,k_{K}\right\}$ using the weighted K nearest neighbors method. To this end, the weights for each node are calculated as:

(8) $$w_{j}=\frac{D_{j}}{\sum_{i=1}^{K}D_{k_{i}}}$$

for $j=\left\{k_{1},\ldots ,k_{K}\right\}$, where K is the number of reference nodes chosen for the second phase. With this result, the final (x, y) position estimation of the localized node is determined as a sum of the weighted positions of the considered K reference nodes:

(9) $$\left(x,y\right)=\left(\overset{k_{K}}{\underset{j=k_{1}}{\sum }}w_{j}x_{j},\;\overset{k_{K}}{\underset{j=k_{1}}{\sum }}w_{j}y_{j}\right)$$

where $\left(x_{n},y_{n}\right)$ are the coordinates of the *j*th reference node.

**Figure 4:**
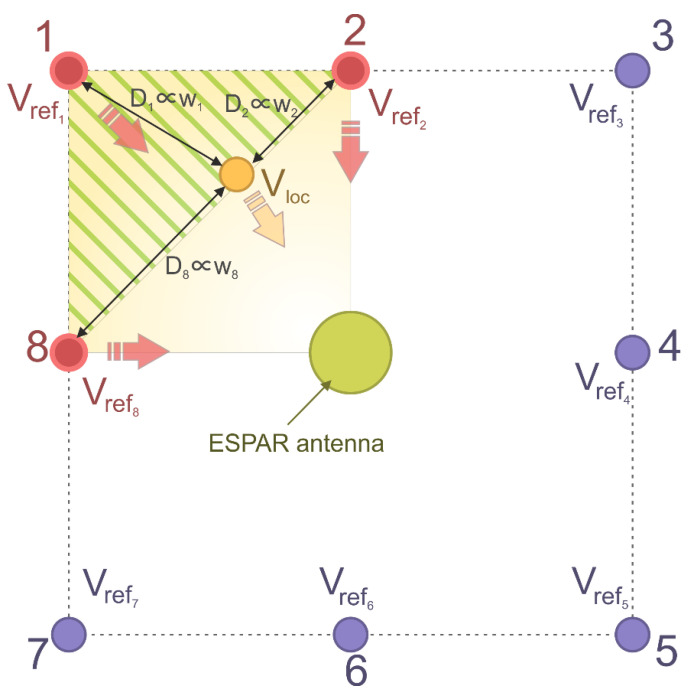
Proposed calibration-free algorithm overview. The position of localized node $V_{loc}$ (marked as a yellow dot) is calculated using 3 reference nodes (*K* = 3, marked in red), for which the associated Euclidean distances $D_{j=k_{2}}<D_{j=k_{1}}<D_{j=k_{8}}$ calculated in the first phase of the estimation are the smallest (see text for explanations).

**Figure 5:**
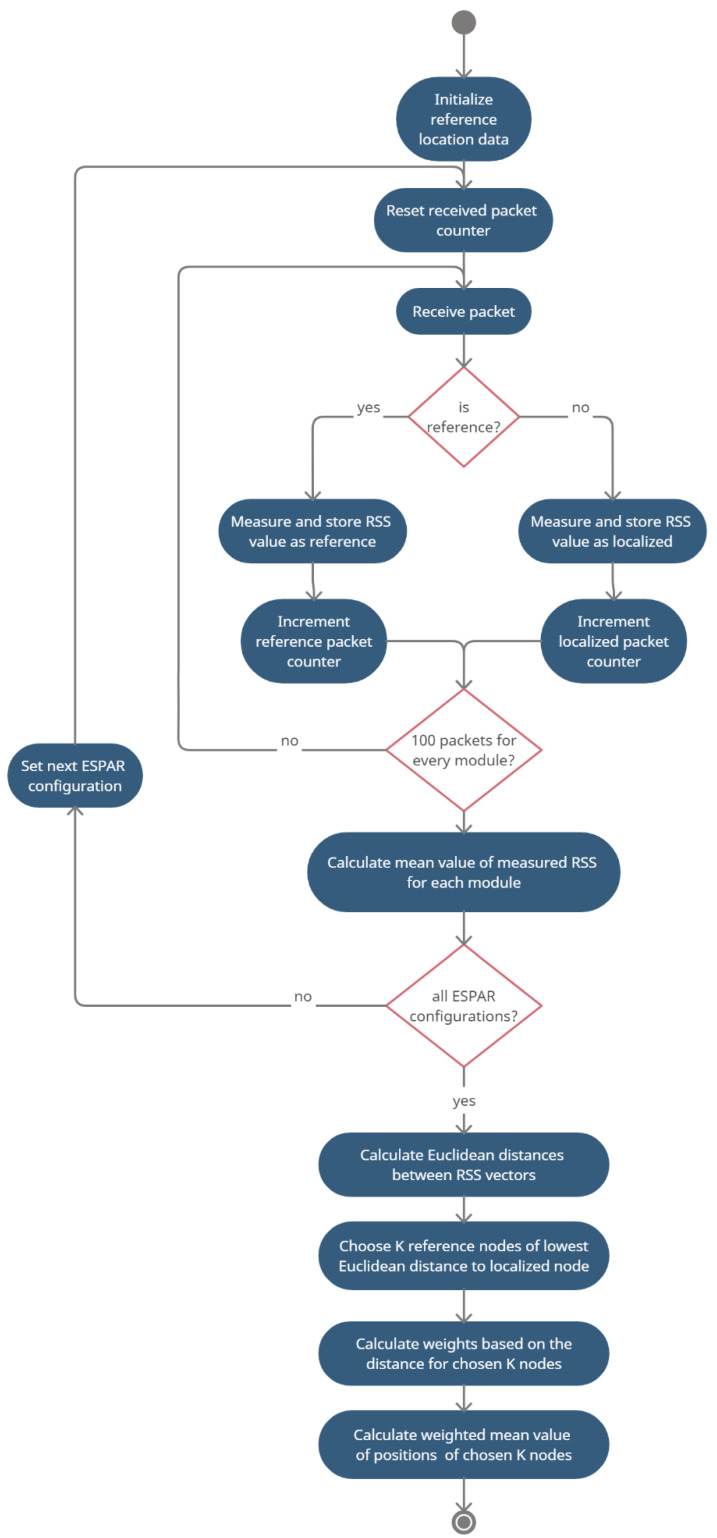
Activity diagram for the proposed calibration-free indoor positioning method (see text for explanations).

An exemplary localization for 8 reference nodes and *K* = 3 is illustrated in Figure 4. The nodes for which the distance *D_j_* is the smallest were chosen for the second phase of the estimation process and are marked in red. Red and yellow arrows represent signals chosen for the estimation. The hatched area represents the potential position of the localized node based on the position of the chosen reference nodes. The final estimation of the localized node position is represented by the orange circle. A complete flow diagram of the position estimation procedure is shown in Figure 5.

## 5. Test Environment

**Figure 6:**
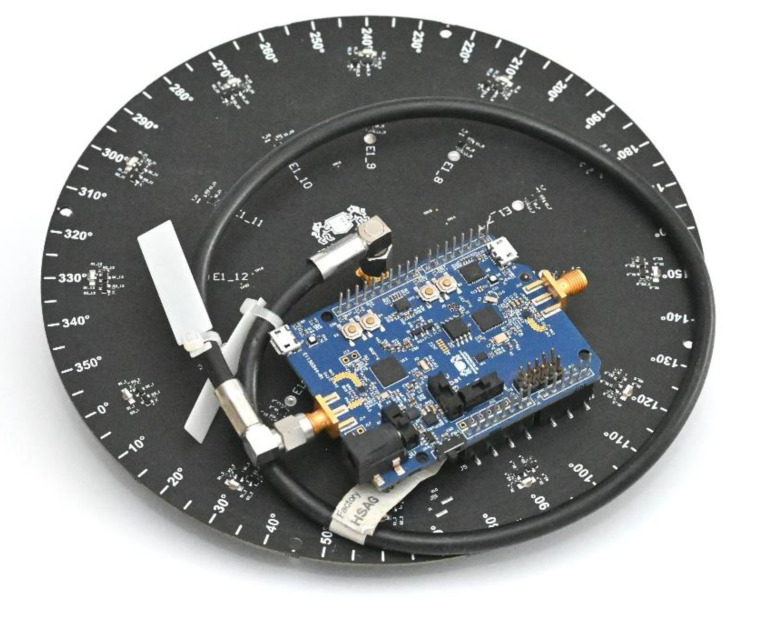
The ESPAR antenna connected to the custom-made nRF52840 wireless sensor network (WSN) board via Arduino Shield header pins. The WSN board’s radio frequency (RF) signal output is connected to the ESPAR antenna’s center element via a black SMA cable.

To verify the proposed calibration-free indoor positioning procedure using single-anchor estimation based on an ESPAR antenna, a dedicated test area and equipment were prepared. For the experiment, the ESPAR antenna was connected to a dedicated, custom-made WSN module based on the Bluetooth Low Energy (BLE)-compliant Nordic nRF52840 SoC. The antenna output of the board was connected to the ESPAR antenna through an SMA connector, while the antenna was steered using GPIO connectors, as shown in Figure 6.

Additionally, 25 BLE Nordic nRF52840 dongles, each with an integrated 2.4 GHz PCB antenna, were prepared as the reference and localized nodes. The nRF52840 SoC is equipped with a 32-bit ARM Cortex-M4F, has output power programmable from +8 dBm to −20 dBm, and has −96 dBm sensitivity. Even though the functionality and computation power of the nRF52840 dongles exceed the requirements for reference nodes, they were chosen to assure the integrity of the system, minimizing the potential influence of hardware diversity on the obtained positioning evaluation results. Nevertheless, in real applications, for the localized and reference devices, simple BLE beacons, which retail for 2–3 USD per item, can be utilized.

**Figure 7:**
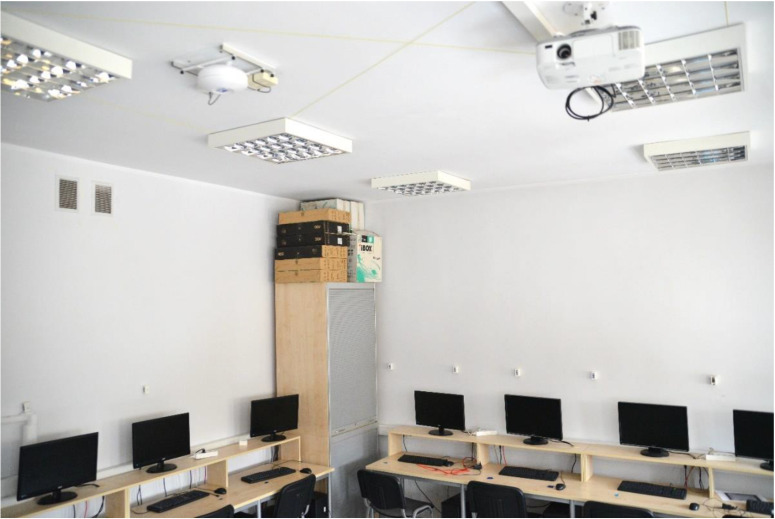
Test environment with reference nodes installed on the walls and the ESPAR antenna integrated with the custom-made WSN board in a single housing mounted on the ceiling.

**Figure 8:**
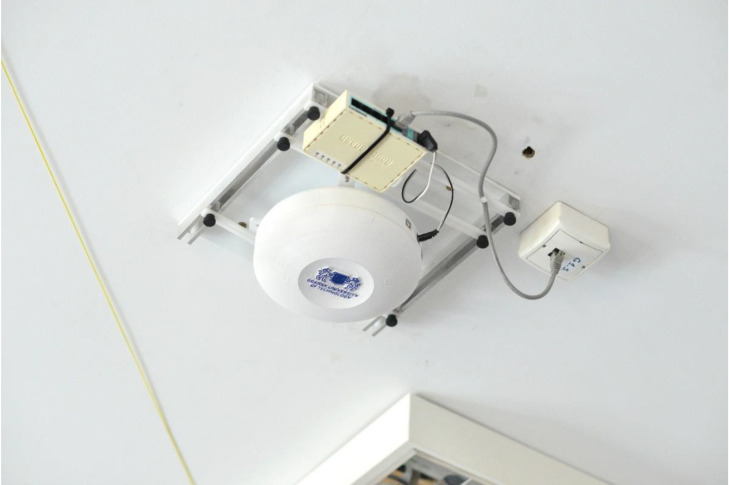
The ESPAR antenna integrated together with the custom-made WSN board installed within a custom-made housing and mounted on the ceiling.

**Figure 9:**
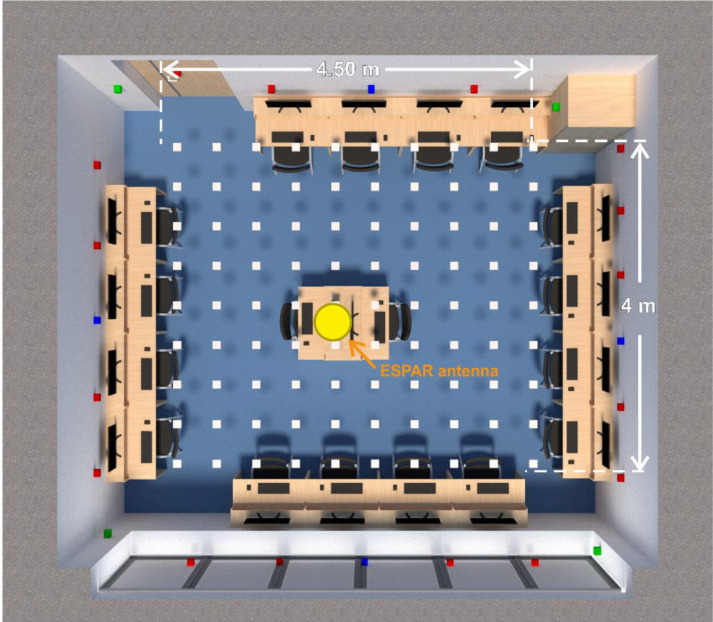
A 3D plan of the test environment, with the test positions marked as white squares and the reference nodes as blue, green, and red squares (see explanations in text).

For the test environment, a 5.6 m × 6.6 m laboratory room was chosen, as the dimensions of this room were most similar to test environments in which other single-anchor positioning methods were evaluated in the literature [26,27]. The base station equipped with the ESPAR antenna was installed on the ceiling in the center of the room, while the 24 reference nodes were placed at even distances on the walls 1.5 m above the floor, which was 1.5 m below the base station, as shown in Figure 7 and Figure 8. The positions of the devices were measured with a precise laser distance measurer and the measuring tape to assure appropriate placement accuracy. The localized node and all of the reference nodes were set into advertising mode and the transmission power was set to 0 dBm. To conduct the measurement session, a 4.5 m × 4.5 m grid was added on the floor with a 0.5 m step, which resulted in 90 test points, as illustrated in Figure 9.

**Figure 10:**
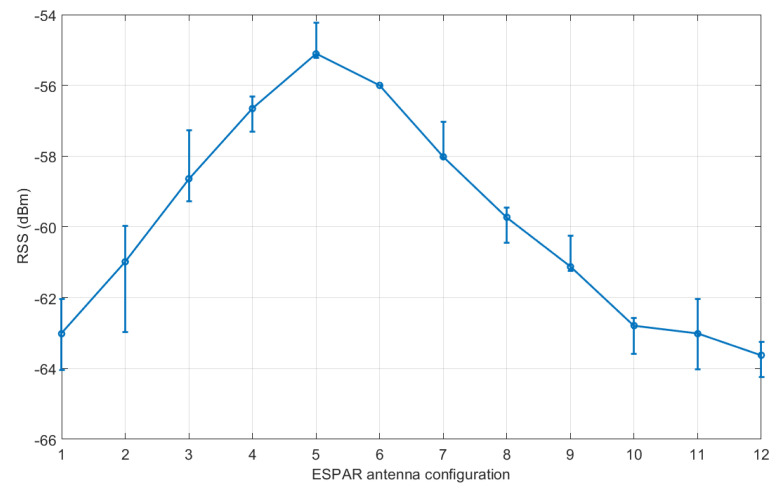
Received signal strength from a single reference module with respect to ESPAR antenna configurations. Error bars represent the maximal received signal strength (RSS) deviation within 100 measurement packets. The standard deviation for RSS values σ=0.316 dB.

Figure 10 presents the RSS values measured in the base station equipped with the ESPAR antenna from a single reference node for each of the 12 antenna configurations. One can easily notice that an approximately 8 dB spread between the maximum and minimum values is present in the measurements. The results show that in the proposed setup, there is noticeable angular diversity between the directions of ESPAR antenna radiation patterns, which is necessary for the proposed single-anchor positioning process. Similar results were obtained for all of the considered reference nodes.

## 6. Measurement Results and Discussion

To evaluate the proposed method, for every grid point, a series of measurements in the aforementioned test environment were conducted. To this end, the localized device was placed at each point of a 4.5 m × 4 m grid shown in Figure 9. At every test point, RSS measurements of 100 transmitted packets were collected for each antenna configuration from each of the reference nodes and the localized node, giving a total of 30,000 randomly collected RSS values for one grid point. To avoid RSS fluctuation between Bluetooth channels, a single channel (number 38) was chosen for the analysis. For each test point, the mean value of the RSS measurements was calculated. Measurements took approximately 20 min for each test point.

**Figure 11:**
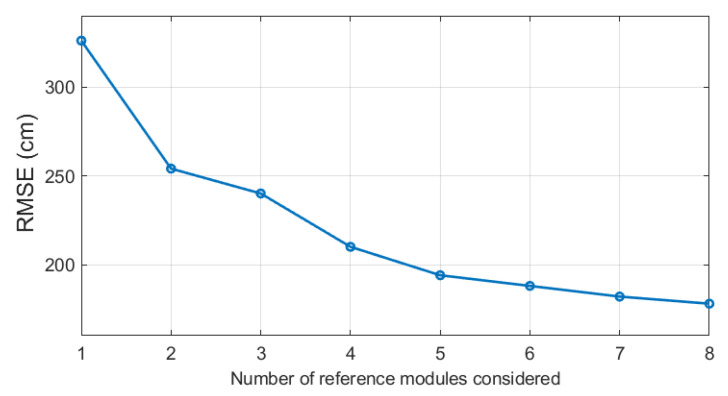
Root mean square error (RMSE) for different numbers of modules of the lowest Euclidean distance considered for the second phase of the proposed localization algorithm.

In Table 2 and Figure 11, the resultant accuracy for different numbers of *K* chosen for the second phase of the estimation are presented. The results indicate that the more reference modules are considered in the algorithm in the second phase, the more accurate the estimation is. However, one can notice that for more than 4 reference modules, the increase of the accuracy is less significant.

To examine how much the number of reference nodes could be reduced, additional tests were performed. To this end, three different layouts for reference nodes were investigated: 4 nodes in the corners of the room (only green reference nodes—see Figure 8), 4 nodes in the middle of each wall (only blue reference nodes), and a total of 8 nodes with a combination of both blue and green reference nodes. For each configuration, the algorithm was set to choose 2, 3, and 4 nodes of the lowest Euclidean distance for the position estimation. The results are presented in Table 3.

The results show that even a significant reduction of the number of reference modules does not affect the accuracy of the estimation. In fact, for every analyzed case with a different number of modules *K*, the accuracy increased when compared to the configuration with 24 reference nodes. Additionally, for each value of *K*, the reduced configurations exhibited very similar accuracy when compared to each other. Thus, it can be concluded that 4 reference nodes per room of a similar size is enough to obtain sufficient position estimation. Nevertheless, one can easily notice that the obtained results are not satisfactory, since both the mean error and maximal error are similar or higher to those that would be calculated with a simple method that always provides estimation in the center of the room (mean error of 1.81 m).

To investigate the possibility for further accuracy improvement, the influence of the RSS normalization, as proposed in [48], on the accuracy was verified. The aim of this modification of RSS values is to mitigate the potential influence of hardware diversity between the reference nodes or uneven distance of the ESPAR antenna from the nodes. To this end, each RSS vector was normalized by rescaling the elements to fall into the range of [0, 1] using the following formula:

(10) $$RSS_{norm}{{}_{ref_{j}}}_{i}=\frac{RSS_{ref_{j_{i}}}-min\left(V_{ref_{j}}\right)}{max\left(V_{ref_{j}}\right)-min\left(V_{ref_{j}}\right)}$$

where $V_{ref_{j}}=\left\{RSS_{ref_{j_{1}}},\;\ldots ,RSS_{ref_{j_{12}}}\right\}$ for the *j*th reference node, $RSS_{norm}{{}_{ref_{j}}}_{i}\in \left[0,1\right]$, and:

(11) $$RSS_{norm_{loc_{i}}}=\frac{RSS_{loc_{i}}-min\left(V_{loc}\right)}{max\left(V_{loc}\right)-min\left(V_{loc}\right)}\;$$

where $V_{loc}=\left\{RSS_{loc_{1}},\;\ldots ,RSS_{loc_{12}}\right\}$ and $RSS_{norm_{loc_{i}}}\in \left[0,1\right]$. In consequence, the Euclidean distances rely only on the information regarding the direction of each node and can be calculated as:

(12) $$D_{norm}{}_{j}=\sqrt{\overset{12}{\underset{i=1}{\sum }}{\left(RSS_{norm_{loc_{i}}}-RSS_{norm}{{}_{ref_{j}}}_{i}\right)}^{2}}$$

The accuracy results obtained for normalized RSS vectors are shown in Table 4.

It can be noticed that for almost all of the considered configurations, the RSS normalization provides a further increase of accuracy as compared to the results in Table 3. The best accuracy is achievable for 4 reference nodes and K = 3 is used in the second phase of the algorithm, resulting in a mean localization error of 1.39 m, indicating that this configuration is optimal for this method. As shown in Table 5, the reference nodes installed in the middle of each wall provide 13.7% higher accuracy when compared to the fingerprinting ESPAR-antenna-based approach presented in [26], which resulted in a mean localization error of 1.61 m for measurements conducted in a room of a similar size. Additionally, the proposed system provides 17.7% higher accuracy when compared to the single-anchor DoA-based approach presented in [27] for a switched-beam antenna, where measurements were performed in an area measuring 7.2 m × 8 m with 24 test points, resulting in a mean localization error of 1.69 m.

**Figure 12:**
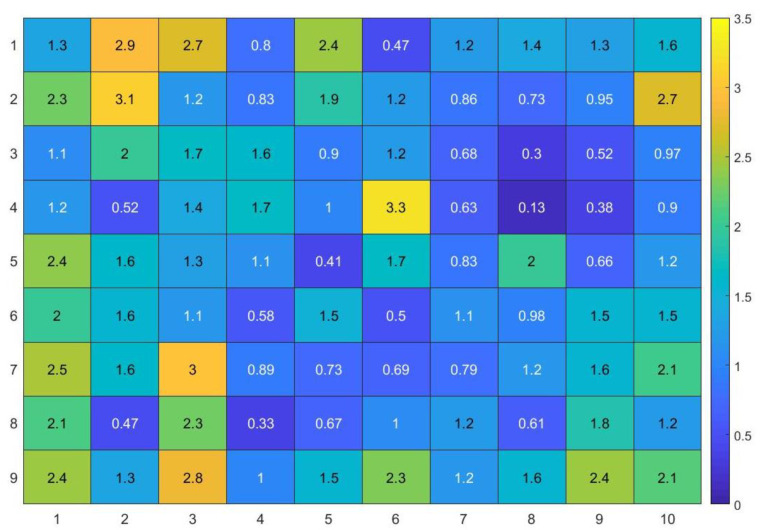
Estimation error (m) calculated for each test point.

**Figure 13:**
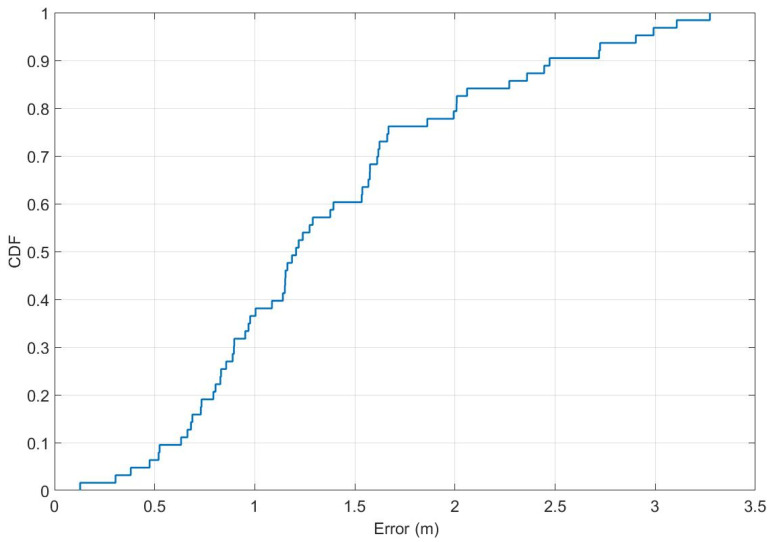
Localization errors in the form of a cumulative distribution function.

Figure 12 and Figure 13 present further analyses of the obtained results for individual test points. One can notice that high error values occur only incidentally, and in most cases near the walls or the furniture.

To verify the reproducibility of the estimation results and the stability of RSS measurements, for each test point, the collected measurements were respectively divided into 2, 3, 5, 10, 20, and 50 equal datasets of 50, 30, 20, 10, 5, and 2 RSS values measured from each node. For each dataset, position estimation was performed and the results were compared with the results achieved for the full dataset of 100 RSS values. Additionally, the approximated time difference between the first and the last estimation and the standard deviation of the mean estimation error were calculated. As shown in Table 6, even for the estimation where 2 packets per node were used, the obtained results were stable and very similar to those for the mean value calculated with the full dataset of 100 packets per node.

## 7. Conclusions

In this paper, a calibration-free single-anchor indoor localization method, including a dedicated algorithm and all necessary hardware modules, has been proposed. It has been shown that a single WSN base station equipped with an ESPAR antenna to perform the measurements can be used to find the position of an unknown BLE tag without calibration or recalibration, since inexpensive reference modules installed on walls within the test area provide enough reference information for the positioning algorithm. An analysis of different layouts of reference modules indicated that the use of only 4 modules is sufficient for the method to provide reliable results. Moreover, optional normalization of RSS vectors allows further increases of the accuracy. Since the algorithm does not need high computational power, it can be implemented in a simple WSN sensor, which additionally contributes to the system’s cost effectiveness. Measurements in a real-life 5.6 m × 6.6 m environment have shown that the proposed single-anchor approach employing an ESPAR antenna in a base station mounted on the ceiling is applicable in real-life scenarios, achieving a mean accuracy of 1.39 m, which is approximately 13.7% better than the accuracy of the fingerprinting method (1.61 m) reported previously with a similar setup and antenna [26] and approximately 17.7% better than the single-anchor approach presented in [27], both of which additionally require much a more time-consuming deployment calibration and regular recalibrations to adjust to changing propagation environments. Furthermore, the obtained results are reproducible and can be achieved even for a significantly reduced number of RSS measurements. As a consequence, the proposed calibration-free single-anchor indoor localization method using an ESPAR antenna can be effectively implemented in real, cost-efficient deployments, providing reliable position estimation.

## Figures and Tables

**Table 1:** The antenna’s main beam directions for different steering vectors applied to the Electronically Steerable Parasitic Array Radiator (ESPAR) antenna.

n	$\varphi_{max}^{n}$	$V_{max}^{n}$
1	90°	111110000000
2	120°	011111000000
3	150°	001111100000
4	180°	000111110000
5	210°	000011111000
6	240°	000001111100
7	270°	000000111110
8	300°	000000011111
9	330°	100000001111
10	0°	110000000111
11	30°	111000000011
12	60°	111100000001

**Table 2:** The estimation accuracy results (maximum error, mean value, and root mean square error (RMSE)) calculated for varying numbers of reference modules considered for the second phase of the proposed localization algorithm.

K	Max. Error (m)	Mean Error (m)	RMSE (m)
3	5.36	2.16	2.40
4	4.51	1.87	2.10
5	3.93	1.67	1.94
8	3.95	1.54	1.78

**Table 3:** Estimation accuracy of the proposed localization algorithm, including the maximum error, mean value, and root mean square error (RMSE) calculated for various limited layouts of reference modules.

K	Reference Modules Configuration	Max. Error (m)	Mean Error (m)	RMSE (m)
2	corners (4 modules)	4.89	2.22	2.46
	middle (4 modules)	4.89	2.02	2.24
	corners and middle (8 modules)	5.52	2.19	2.49
	all (24 modules)	5.42	2.29	2.54
3	corners (4 modules)	4.09	1.82	2.05
	middle (4 modules)	3.68	1.97	2.14
	corners and middle (8 modules)	3.97	1.98	2.18
	all (24 modules)	5.36	2.16	2.40
4	corners (4 modules)	3.98	1.72	1.85
	middle (4 modules)	3.85	1.74	1.86
	corners and middle (8 modules)	4.64	1.88	2.14
	all (24 modules)	4.51	1.87	2.10

**Table 4:** The overall estimation accuracy results (maximum error, mean value, and RMSE) calculated with received signal strength (RSS) normalization introduced in the proposed localization algorithm.

K	Reference Modules Configuration	Max. Error (m)	Mean Error (m)	RMSE (m)
2	corners (4 modules)	5.04	2.42	2.62
	middle (4 modules)	4.06	1.63	1.87
	corners and middle (8 modules)	5.09	2.36	2.59
	all (24 modules)	4.21	2.18	2.38
3	corners (4 modules)	3.95	1.63	1.82
	middle (4 modules)	3.58	1.39	1.58
	corners and middle (8 modules)	3.52	1.84	2.04
	all (24 modules)	3.90	1.99	2.18
4	corners (4 modules)	3.58	1.68	1.82
	middle (4 modules)	4.13	1.63	1.76
	corners and middle (8 modules)	3.93	1.55	1.77
	all (24 modules)	3.84	1.86	2.04

**Table 5:** Comparison of accuracy results for the presented approach and other single-anchor indoor positioning methods.

Positioning Method	Mean Error (m)	Calibration Needed
Calibration-free single-anchor	1.39	NO
Single-anchor fingerprinting [26]	1.61	YES
Single-anchor DoA [27]	1.69	YES

**Table 6:** Deviation of mean estimation error results calculated for a reduced number of RSS values.

Number of Packets	50	30	20	10	5	2
Number of estimations for each test point	2	3	5	10	20	50
Approximated time difference between the first and last estimation [min]	10	12	16	18	19	19.6
Maximal difference of mean estimation error when compared to full dataset [m]	0	0.01	0.01	0.01	0.03	0.07
Standard deviation of mean error [m]	0.0001	0.0004	0.001	0.002	0.008	0.01

## Data Availability

Not applicable.
